# A *Shigella flexneri* 2a synthetic glycan-based vaccine induces a long-lasting immune response in adults

**DOI:** 10.1038/s41541-023-00624-y

**Published:** 2023-03-10

**Authors:** Shiri Meron-Sudai, Valeria Asato, Amos Adler, Anya Bialik, Sophy Goren, Ortal Ariel-Cohen, Arava Reizis, Laurence A. Mulard, Armelle Phalipon, Dani Cohen

**Affiliations:** 1 School of Public Health, Sackler Faculty of Medicine, Tel Aviv University, Ramat Aviv, Tel Aviv, 69978 Israel; 2 Clinical Microbiology Laboratory, Tel-Aviv Sourasky Medical Center, 6 Weizmann Street, Tel Aviv, 6423906 Israel; 3 Institut Pasteur, Université Paris Cité, CNRS UMR3523, Unité de Chimie des Biomolécules, F-75015 Paris, France; 4 Institut Pasteur, Innovation Lab. Vaccines, F-75015 Paris, France

**Keywords:** Drug development, Vaccines

## Abstract

*Shigella* is a leading cause of moderate to severe diarrhea worldwide and of diarrhea-associated deaths in children under 5 years of age in low-and middle-income countries. A vaccine against shigellosis is in high demand. SF2a-TT15, a synthetic carbohydrate-based conjugate vaccine candidate against *Shigella flexneri* 2a (SF2a) was found safe and strongly immunogenic in adult volunteers. Here, SF2a-TT15 at 10 µg oligosaccharide (OS) vaccine dose is shown to induce a sustained immune response in magnitude and functionality in the majority of volunteers followed up 2 and 3 years post-vaccination. High levels of either one of the humoral parameters as well as the number of specific-IgG memory B-cells determined 3 months after vaccination were good predictors of the durability of the immune response. This study is the first to examine the long-term durability of antibody functionality and memory B-cell response induced by a *Shigella* vaccine candidate.

## Introduction

Bacteria of the genus *Shigella* are a leading cause of moderate to severe diarrhea and the main cause of dysentery worldwide^1,2^. The greatest burden of shigellosis is in low-and middle-income countries (LMICs) with an estimate of 250 million cases of disease and over 212,000 deaths per year at all ages, 63,000 of which are among children under 5 years of age^1,2^. *Shigella* infection is associated with malnutrition^1,3^, persistent and prolonged diarrhea^4^ and linear growth faltering^5,6^. Around 1.5–2 million cases of shigellosis also occur annually in high-income countries where at-risk populations are children below 5 years of age living in crowded communities, soldiers serving under field conditions, men who have sex with men, and travelers to LMICs^1,7–11^. Hygiene measures often fail to prevent the transmission of *Shigella* due to the very low inoculum of only 100–1000 organisms that are required to cause shigellosis^12^. In severe cases of shigellosis antibiotic treatment is indicated, however, the increase in antimicrobial resistance of *Shigella* reduces the antibiotic treatment options^9,13,14^. There are four species or serogroups of *Shigella*: *S. dysenteriae*, *S. flexneri*, *S. boydii* and *S. sonnei*, which comprise more than 50 different serotypes. *S. flexneri* accounts for most cases of shigellosis in LMICs and *S. sonnei* is responsible for the majority of cases in high-income countries^15^. The high burden of shigellosis, the increasing resistance of *Shigella* spp. to antibiotics and the sanitation and hygiene conditions in LMICs, have made the development of *Shigella* vaccines a recognized priority by the World Health Organization^16^. Despite many decades of research and several vaccine candidates in the pipeline, there is still no licensed vaccine available to prevent shigellosis.

*Shigella* infection confers serotype-specific immunity indicating that the O-specific polysaccharide (O-SP) is the protective antigen^7,17,18^. Live-attenuated vaccine strains and inactivated *Shigella* vaccine candidates have been major strategies to orally deliver the protective O-SP antigen. Yet, the narrow window between immunogenicity and reactogenicity and the discrepancy in the potential protective capacity when tested in western volunteers and in individuals in endemic areas slowed down significantly the process of development of promising live, rationally attenuated oral vaccine candidates^18,19^. A breakthrough was achieved in the *Shigella* vaccine field by the parenterally delivered detoxified *Shigella* O-SP antigen conjugate vaccines, developed at the National Institutes of Health by John B. Robbins and Rachel Schneerson^20,21^. Efficacy was originally demonstrated for a *S. sonnei* detoxified LPS-recombinant exotoxin A from *Pseudomonas aeruginosa* (dLPS-rEPA) conjugate in young Israeli soldiers^22^ and subsequently in Israeli children down to 3 years of age^23^. These studies were critical for establishing serum IgG anti-LPS as a correlate of protection against shigellosis^24,25^. Moreover, they guided the development of a diversity of second generation injectable mono- and multivalent glycoconjugates and of other O-SP-based *Shigella* vaccine candidates aiming to generate protective immunity in the target population of infants in LMICs. Four candidates are currently in advanced clinical development^19^. *S. flexneri* 2a (SF2a) bioconjugate vaccine (Flexyn2a) was safe, immunogenic and demonstrated protective efficacy against moderate to severe SF2a shigellosis in a controlled human infection model (CHIM) study^26,27^. The current quadrivalent configuration of the vaccine (SF2a, 3a, 6 and *S. sonnei*) designed to cover the vast majority of *Shigella* serotypes worldwide, is completing a phase II study in Kenyan infants^28^. Alternatively, the 1790GAHB *S. sonnei* vaccine candidate uses the generalized modules for membrane antigens (GMMA) platform to deliver the *Shigella* O-SP. This GMMA candidate was found safe and immunogenic in clinical trials in non-endemic and endemic countries, but did not protect against *S. sonnei* shigellosis in a CHIM study^29,30^. At present, a quadrivalent combination of the GMMA vaccine candidates, that includes *S. flexneri* 1b, 2a, 3a, and *S. sonnei*, with increased O-antigen dosages is in clinical evaluation in European adults before moving to a phase IIa study in LMIC young children and infants^31^. Otherwise, the *Shigella* invasin complex vaccine (Invaplex) candidate is a type of subunit vaccine that contains *Shigella* LPS and the invasion plasmid antigen (Ipa) proteins B and C. Developed for parenteral administration, the most recent detoxified artificial Invaplex (Invaplex_AR-Detox_), has demonstrated a good safety and immunogenicity profile in a phase I study in North American volunteers and is candidate toward evaluation in the target populations of children in LMICs^32^.

Lastly and notably differing from the above by its glycan component, the SF2a-TT15 candidate vaccine is the prototype of synthetic carbohydrate-based conjugate vaccine candidates developed at Institut Pasteur. It features a chemically synthesized 15-mer oligosaccharide (OS) hapten^33^, linked through its reducing terminus via single-point attachment onto tetanus toxoid (TT) in an average OS:TT molar ratio of 17^34^. SF2a-TT15 conjugate was developed as a monovalent vaccine candidate against SF2a^35^, one of the prevalent *Shigella* serotypes^15^. The selected fine-tuned OS corresponds to a three basic repeating unit segment of the O-SP component of the LPS of SF2a and was shown to act as a structural and antigenic surrogate thereof^33,35–37^. Pre-clinical studies showed long-lasting protective SF2a LPS-specific-IgG antibodies in mice immunized with SF2a-TT15^34,35^. Moreover, they revealed that SF2a-TT15-induced antibodies recognize a large panel of SF2a circulating strains^38^. Following the successful production of a GMP (Good Manufacturing Practice) batch of SF2a-TT15^38^, a first in human phase I study was conducted during 2016-2017 at the Clinical Research Center, Tel Aviv Sourasky Medical Center and the Tel Aviv University School of Public Health^39^. The study design together with the safety and immunogenicity outcomes were described in detail elsewhere^39^. Briefly, in this dose-escalating, single-blind, randomized, placebo-controlled study, 64 healthy adult volunteers (aged 18–45 years) were enrolled and assigned to receive either the low dose of the vaccine candidate, equivalent to 2 μg OS amount, or the high dose, equivalent to 10 μg OS amount, adjuvanted or not with aluminum hydroxide (alum), or matching placebo. Three single intramuscular injections were administered 28 days apart and participants were followed up for 3 months after the last injection. SF2a-TT15 was safe and well tolerated. It induced a strong immune response as measured by serum IgG and IgA antibody levels, serum bactericidal activity (SBA), antibody avidity, antibody-secreting cells, memory B-cells, serum IgG antibody subclasses, IgA urinary antibodies and antibodies in lymphocyte supernatant^39^.

Here, we report the findings of a long-term follow-up study among the volunteers of phase I clinical trial to assess the longevity of the immune response induced by the SF2a-TT15 vaccine candidate, 2 and 3 years after vaccination.

## Results

SF2a-TT15 glycoconjugate was safe and well tolerated in the first-in-human, dose-escalating phase I study. Both low (2 µg OS) and high (10 µg OS) doses of the vaccine candidate were immunogenic by means of all quantitative and functional parameters tested with stronger response induced by the high dose, regardless of adjuvanted or non-adjuvanted formulations. After one injection, the non-adjuvanted 10 μg OS dose induced a 27-times increase in IgG Geometric Mean Titer (GMT) and the non-adjuvanted 2 μg OS dose induced a five-times increase, compared with baseline. The second and third injections did not further increase the immune response that has remained stable when measured 3 months later, at the end of the phase I study^39^.

### High levels of anti-SF2a LPS serum IgG and IgA persist for 3 years post-vaccination

**Fig. 1:**
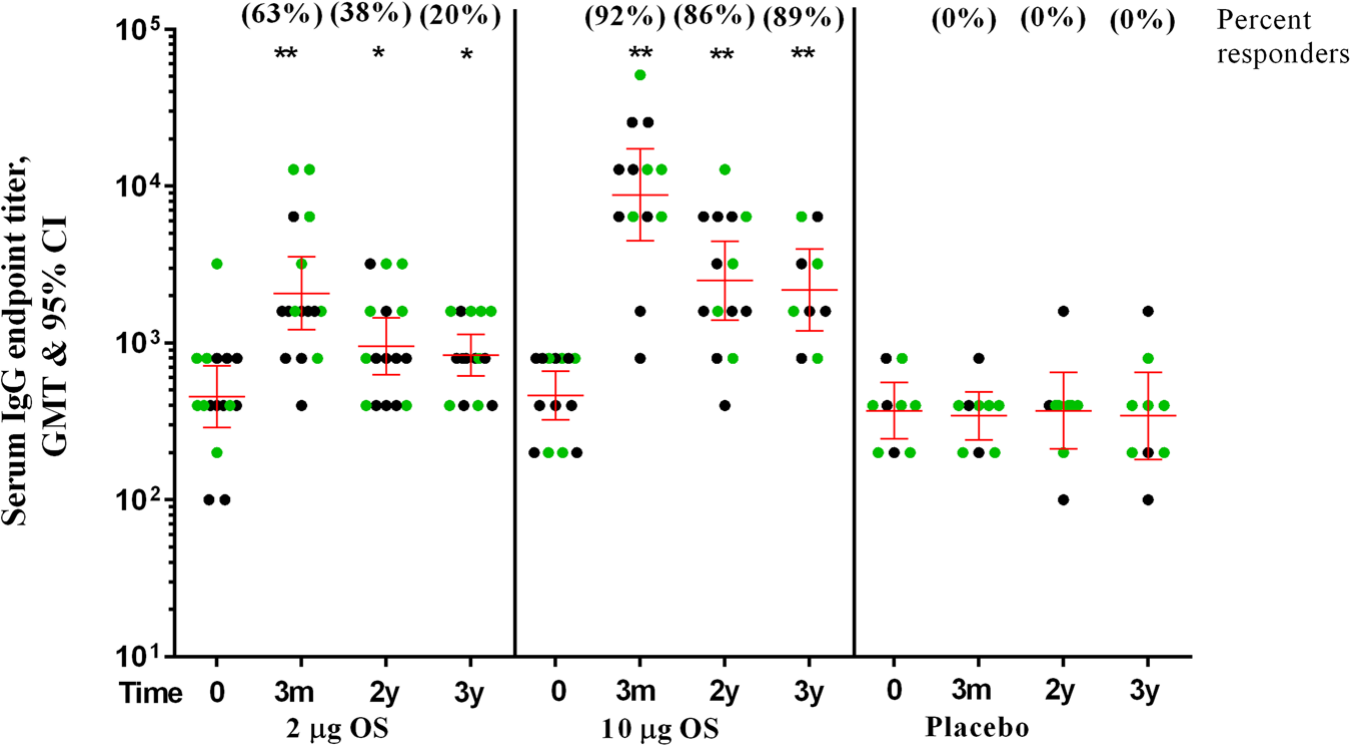
Longevity of serum IgG GMT (95% CI) to SF2a LPS in vaccinees receiving 2 or 10 µg OS doses of non-adjuvanted and adjuvanted SF2a-TT15 and in placebo recipients. Circles represent individual endpoint titres, green circles represent volunteers receiving the adjuvanted vaccine or placebo. Bars represent the Geometric Mean Titer (GMT) and the 95% Confidence Intervals (CIs) on day 0 (baseline), at 3 months, 2 and 3 years post-last vaccination. *p-value < 0.05; **p-value < 0.01 (vs. day 0).

GMTs levels of serum IgG decayed by 2 fold and by 3.5 fold in recipients of the 2 µg OS vaccine dose and of the 10 µg OS vaccine dose, respectively, between the last measurement of the phase I study (3 months after last vaccination) and 2 years post-vaccination (Fig. 1 and Supplementary Table 1). There was no significant decrease in GMTs between 2 and 3 years after immunization (Fig. 1 and Supplementary Table 1). As compared to the pre-vaccination levels, the GMT levels of anti-SF2a LPS IgG antibodies were still approximately twice higher in recipients of the 2 µg OS vaccine dose and five times higher in recipients of the 10 µg OS vaccine dose (*p* < 0.05 and *p* < 0.01 for the 2 µg OS and 10 µg OS vaccinees, respectively,Wilcoxon signed rank test) (Fig. 1 and Supplementary Table 1). Three years after vaccination, 20% and 89% of volunteers receiving three injections of the 2 µg OS vaccine dose or 10 µg OS vaccine dose, respectively, maintained a 4-fold or higher titer of anti-SF2a LPS IgGs as compared to their pre-vaccination levels.

The long-term kinetics of the anti-SF2a LPS serum IgA levels had a similar pattern to that observed for serum IgG. There was a 1.6-fold and 2-fold decrease in anti-SF2a LPS IgA GMTs levels 2 years post-vaccination compared to 3 months post-vaccination in recipients of the 2 µg OS vaccine dose and of the 10 µg OS vaccine dose, respectively (Supplementary Figure 1 and Supplementary Table 1). The GMTs were still significantly higher 2 and 3 years after vaccination in the recipients of both doses as compared to the pre-vaccination levels (*p* < 0.01 and *p* < 0.05 for 2 and 3 years, respectively, Wilcoxon signed rank test), though at lower magnitude compared to those of IgG (Supplementary Fig. 1 and Supplementary Table 1). The high dose of vaccine induced a more durable anti-SF2a LPS IgA response than the lower dose as shown by higher GMTs and percent of responders (maintaining a 4-fold or higher increase in antibody titers) 2–3 years after vaccination (Supplementary Fig. 1 and Supplementary Table 1).

Considering the limited number of volunteers reaching the 2 and 3 years immunological follow up, we could not detect statistically significant differences in the longevity of the IgG or IgA levels between the adjuvanted versus the non-adjuvanted formulations for any of the two vaccine doses (Fig. 1, Supplementary Fig. 1 and Supplementary Table 1). No change in GMTs of serum IgG and IgA antibodies to SF2a LPS was observed in the placebo recipients along the follow-up period (Fig. 1, Supplementary Fig. 1 and Supplementary Table 1**)**.

### Functionality of SF2a-TT15 vaccine-induced antibodies is maintained 3 years after vaccination

**Fig. 2:**
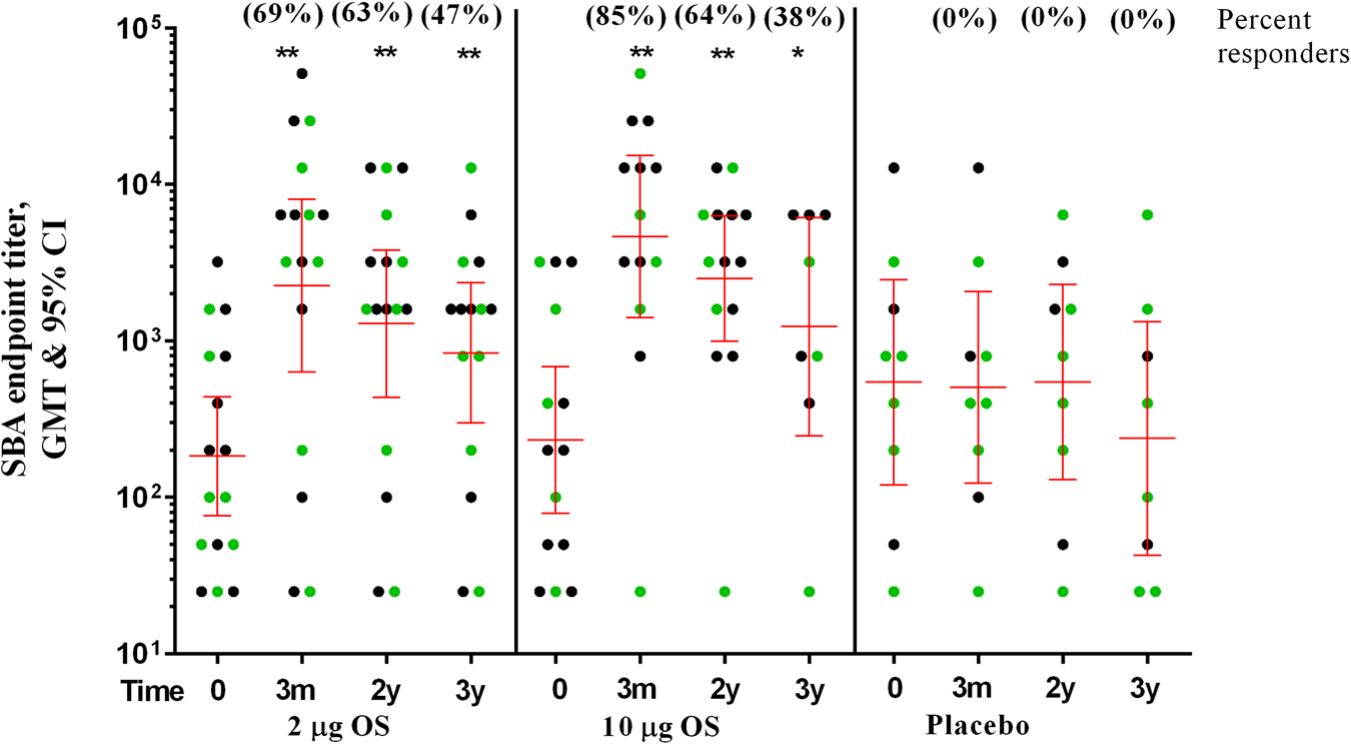
Longevity of SBA GMT (95% CI) in vaccinees receiving 2 or 10 µg OS doses of non-adjuvanted and adjuvanted SF2a-TT15 and in placebo recipients. Circles represent individual endpoint titers, green circles represent volunteers receiving the adjuvanted vaccine or placebo. Bars represent the geometric mean titres (GMTs) and the 95% Confidence Intervals (CIs) on day 0 (baseline), at 3 months, 2 and 3 years post-vaccination. Serum Bactericidal Activity (SBA): a serum dilution yielding at least 50% reduction in the number of colonies compared with control was defined as having bactericidal activity. The last serum dilution with bactericidal activity was defined as the endpoint titer. *p-value < 0.05; **p-value < 0.01 (vs. day 0).

Two years post-vaccination a decline of less than 2-fold was observed in SBA GMTs in both groups of vaccinees when compared to 3 months post-vaccination (Supplementary Table 1). This decay was in line with that observed in the GMTs of anti-SF2a LPS IgG. The GMTs of SBA were still 7- and 11-fold higher than the pre-vaccination levels for volunteers receiving the 2 µg and 10 µg OS doses, respectively (Fig. 2 and Supplementary Table 1). At 3 years post-immunization, SBA GMTs were still of approximately 5-fold-higher than baseline levels, for the 2 µg and 10 µg OS doses (Fig. 2 and Supplementary Table 1).

Sixty-three percent of volunteers receiving the 2 µg OS dose and 64% of those receiving the 10 µg OS dose, maintained a 4-fold or higher rise in SBA titers 2 years after vaccination. At 3 years after vaccination the corresponding percentages were 47% and 38% for the 2 µg and 10 µg OS dose recipients, respectively (Supplementary Table 1). No significant changes were observed in the SBA GMTs of placebo recipients during the 3 years of follow up (Fig. 2 and Supplementary Table 1).

**Fig. 3:**
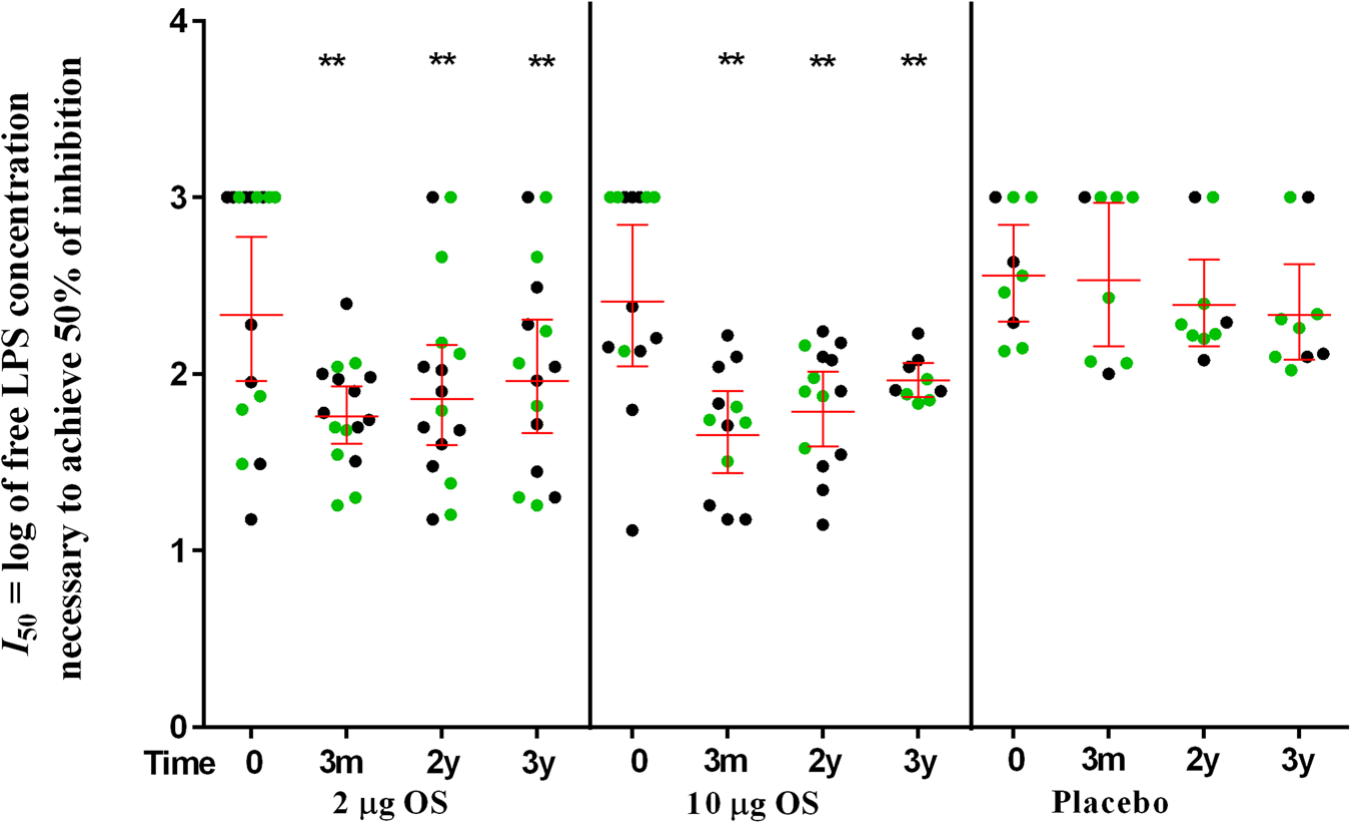
Longevity of Mean Avidity *I*_*50*_ (95% CIs) of IgG serum antibodies to SF2a LPS in vaccinees receiving 2 or 10 µg OS doses of non-adjuvanted and adjuvanted SF2a-TT15 and in placebo recipients. Circles represent individual *I*_*50*_ index, green circles represent volunteers receiving the adjuvanted vaccine or placebo. Bars represent Mean *I*_*50*_ and the 95% Confidence Intervals (CIs) on day 0 (baseline), at 3 months, 2, and 3 years post-vaccination. **p-value < 0.01 (vs. day 0).

Despite the somewhat decline observed from the last measurement performed 3 months after last vaccination, the avidity of IgG anti-SF2a LPS IgG antibodies remained significantly higher (lower *I*_50_) compared to baseline levels, for both doses, 2 and 3 years after the last immunization (*p* < 0.01, Wilcoxon signed rank test) (Fig. 3 and Supplementary Table 1). No significant variations in antibody avidity were observed in placebo recipients along the 3 years of follow up (Fig. 3 and Supplementary Table 1).

As shown in Table 1, there was a good to an excellent correlation between the levels of the humoral parameters measured 3 months after the 3rd immunization (the last measurement in the phase I study) with those measured 2 and 3 years later. High levels of either of the humoral parameters determined 3 months after vaccination were good predictors of durability of the immune response 2 and 3 years later (Table 1).

**Table 1:** Spearman’s correlations between the levels of the humoral immune response parameters 3 months and 2 and 3 years after vaccination, in recipients of 2 or 10 µg OS doses of SF2a-TT15 (non-adjuvanted and adjuvanted).

	Serum IgG at 3 months	Serum IgA at 3 months	SBA at 3 months	Avidity at 3 months
Serum IgG at 2 years	*r* = 0.927; *p* < 0.001; *N* = 29			
Serum IgA at 2 years		*r* = 0.872; *p* < 0.001; *N* = 29		
SBA at 2 years			*r* = 0.939; *p* < 0.001; *N* = 29	
Avidity at 2 years				*r* = 0.717; *p* < 0.001; *N* = 28
Serum IgG at 3 years	*r* = 0.876; *p* < 0.001; *N* = 23			
Serum IgA at 3 years		*r* = 0.762; *p* < 0.001; *N* = 23		
SBA at 3 years			*r* = 0.834; *p* < 0.001; *N* = 22	
Avidity at 3 years				*r* = 0.674; *p* = 0.001; *N* = 22

*SBA* serum bactericidal antibodies, r Spearman’s correlation, *N* number of samples.

### IgG and IgA memory B-cell responses to SF2a LPS 2 and 3 years after immunization

**Fig. 4:**
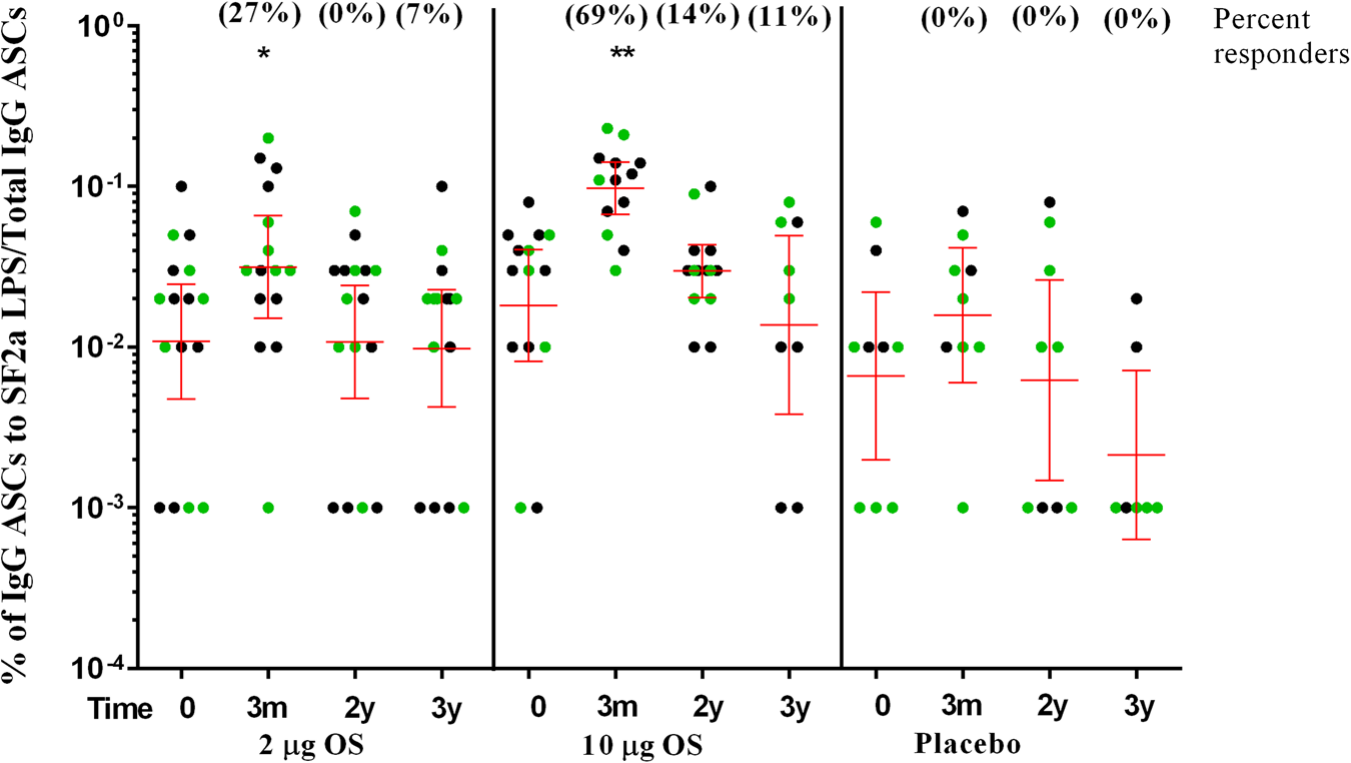
Longevity of IgG memory B-cells to SF2a LPS in vaccinees receiving 2 or 10 µg OS doses of non-adjuvanted and adjuvanted SF2a-TT15 and in placebo recipients. Circles represent individual percent of IgG antibody secreting cells (ASCs) to SF2a LPS /Total IgG ASCs, green circles represent volunteers receiving the adjuvanted vaccine or placebo. Bars represent the Geometric Mean (GM) and 95% Confidence intervals (CIs) of these values on day 0 (baseline), at 3 months, 2 and 3 years post-vaccination. *p-value < 0.05; **p-value < 0.01 (vs. day 0).

The SF2a-TT15 vaccine candidate was shown to induce a marked increase in IgG and IgA memory B-cell specific response to SF2a LPS in the phase I trial^39^. In recipients of the vaccine at high OS dose, a higher magnitude was seen up to 3 months after the last vaccination, as compared to baseline^39^. Two years after vaccination, a steep decline in IgG and IgA memory B-cells was observed in all vaccine recipients as shown by the decrease in the mean percent of SF2a LPS-specific-IgG and IgA memory B-cells toward baseline levels (Fig. 4, Supplementary Fig. 2, and Supplementary Table 2). The decay in the memory B cell response continued in the third year of follow up. For the IgG memory B-cell response to SF2a LPS, 7% and 11% of volunteers receiving the 2 µg and 10 µg OS doses, respectively, were still responders (≥ mean + 2 SD) 3 years after vaccination (Supplementary Table 2). There was no significant change in the levels of IgG and IgA memory B-cell response to SF2a LPS among placebo recipients along the whole follow up period (Fig. 4, Supplementary Fig. 2 and Supplementary Table 2).

### The IgG memory B-cells response to SF2a LPS 3 months after vaccination can predict persistence of the humoral parameters 2 years later

Although IgG and IgA memory B-cell levels returned almost to their baseline 2 years after vaccination, we questioned upon their association at an earlier time point to the humoral response 2 years after vaccination. We examined for all vaccinees (receiving the 2 or 10 µg OS dose) the relationship between “low” and “high” levels of SF2a LPS-specific-IgG and IgA memory B-cells around the geometric mean, 3 months after the last injection (the last measurement of the phase I study)^39^ and the percentage of responders for serum IgG, serum IgA, SBA and mean avidity level 2 years later (Table 2 and Supplementary Table 3). The levels of specific-IgG memory B-cells showed a significant association with the percent of responders for serum IgG (*p* = 0.019, Fisher’s exact test) and serum IgA (*p* = 0.003, Fisher’s exact test), and a border line or trend of association for SBA and mean avidity (*p* = 0.050, Fisher’s exact test and 0.098, Student’s *t* test, respectively) 2 years post-vaccination (Table 2). No such association was found between the levels of SF2a LPS-specific IgA memory B-cells 3 months after last vaccination and the percent of responders for humoral parameters 2 years after vaccination (Supplementary Table 3).

**Table 2:** Frequency of specific-IgG memory B-cells at 3 months post-last vaccination (with 2 or 10 µg OS doses of non-adjuvanted and adjuvanted SF2a-TT15) defined as “low” and “high” values around the geometric mean, and percent of responders (≥4 fold) for serum IgG, serum IgA, SBA and mean value for avidity against SF2a LPS, 2 years after vaccination.

Parameters 2 years after vaccination		Low frequency (≤0.04) *N* = 12	High frequency (>0.04) *N* = 16	*p*-value
Serum IgG	≥4-fold	4 (33.3%)	13 (81.3%)	0.019^a^
Serum IgA	≥4-fold	0 (0.0%)	9 (56.3%)	0.003^a^
SBA	≥4-fold	5 (41.7%)	13 (81.3%)	0.050^a^
Avidity (*I*_50_)	*n* (Mean)	12 (2.06)	16 (1.76)	0.098^b^

Geometric mean of IgG memory B-cells ASC 3 months after vaccination = 0.04.

^a^Fisher’s exact test.

^b^Student’s *t* test.

*SBA* serum bactericidal antibodies, *ASC* antibody secreting cells, *I*_50_ avidity index.

## Discussion

The SF2-TT15 conjugate administered at both low (2 µg OS amount) and high (10 µg OS amount) doses, alum adjuvanted and non-adjuvanted, was safe and strongly immunogenic in the adult volunteers of phase I clinical trial when measured up to 3 months after the third immunization. The 10 µg OS dose was more potent and induced a stronger immune response in magnitude and percentage of responders^39^.

The data generated by the follow-up study indicate that the immune response induced by SF2a-TT15 persisted 3 years after vaccination in terms of both the level and the functionality of antibodies to SF2a LPS. Though decrease after 2 and 3 years as compared to those measured approximately 3 months post-last vaccination, GMTs of serum IgG and IgA remained significantly higher as compared to the pre-vaccination level (*p* < 0.05, Wilcoxon signed rank test). A robust persistence was observed for the higher dose. Whether the adjuvanted formulation, as compared to non-adjuvanted, provided a better longevity of the examined responses remains unknown. Indeed, the number of volunteers reaching the 2 and 3 years immunological follow-up was too limited to detect statistically significant differences. The persistence of serum IgG levels 2 and 3 years following vaccination with SF2a-TT15 can be compared with the corresponding follow-up results shown for the first generation of the *Shigella* dLPS conjugate vaccines^40–42^. In a clinical trial conducted in young adults, immunization with a conjugate vaccine candidate featuring the SF2a dLPS randomly attached to rEPA (SF2a dLPS-rEPA), induced elevated SF2a-specific-IgG and IgA antibody levels when administered as one single injection or two injections six weeks apart at a 25 µg carbohydrate equivalent dose. Although a decrease in GMT over 2 years of followup was observed, the antibody titers remained significantly higher than at pre-vaccination stage^41,42^. Not unexpectedly, a long-lasting serum IgG response was also demonstrated for the non-adjuvanted improved SF2a dLPS-rEPA_succ_ vaccine candidate in adult volunteers (assessed around 6 months at one or two immunization regimen)^43^. More critically, this observation was also valid in the 1–4 years old children^40^ 2 years after vaccination. Overall, the SF2a-specific-IgG response induced by SF2a-TT15 following 3 injections of a 10 µg OS dose, was more durable than that induced by one or 2 injections of either one of the SF2a dLPS-rEPA conjugate or the improved SF2a dLPS-rEPA_succ_ conjugate in terms of percentage of responders 2 years following vaccination (86%, 28%, and 58% for SF2a-TT15, SF2a dLPS-rEPA, and SF2a dLPS-rEPA_succ_ conjugate vaccines, respectively)^40,42^.

Similar to the first generation of the *Shigella* dLPS conjugates, the synthetic carbohydrate-based conjugate, SF2a-TT15, induces a stronger and longer lasting anti-SF2a LPS IgG and IgA response than natural *Shigella* infection as we can assess comparing data generated by studies in which the same ELISA protocols, albeit with slight modifications over the years, were used to measure these parameters^39,44^.

Assessing the functionality of SF2a LPS-specific antibodies is an important tool for determining the mechanistic capabilities of anti-*Shigella* LPS serum IgG as a correlate of protection against shigellosis in the process of a *Shigella* candidate vaccine development^24,25^. The functionality of the specific SF2a-TT15-induced antibodies evidenced in the phase I study up to 3 months after the third and last vaccination^39^ was long-lasting as documented by the avidity and SBA results in sera of volunteers examined 2 and/or 3 years following vaccination. The SF2a-TT15 phase I study was the first to report on a significant increase in avidity of specific anti-*Shigella* antibodies after immunization with a *Shigella* vaccine. The avidity of the anti-SF2a LPS antibodies induced by the SF2a-TT15 conjugate remained significantly higher compared to baseline levels, for both vaccine doses (*p* < 0.01, Wilcoxon signed rank test). The increase in avidity, defined as the strength of the multivalent interaction between antibodies and their antigens, was proposed as a surrogate marker of successful priming after immunization with other conjugate vaccines, in particular licensed ones, like the meningococcal, pneumococcal and *Haemophilus influenzae* type b conjugates^45–47^. Regarding SBA, the antibody-mediated complement-dependent bacteriolysis was proposed as the most plausible mechanistic pathway by which anti-*S. sonnei* LPS IgGs can protect against *Shigella* infection^20^. It has been hypothesized that high levels of serum IgG antibodies induced by *Shigella* conjugate vaccines transuding to the gut mucosal layer together with complement proteins also present on the epithelial surface will lyse the inoculum of *Shigella* at the gut mucosa layer^20^. It is noteworthy that SBA is recognized as a correlate of protection against meningococcal meningitis and that it has been employed to evaluate the protective efficacy of meningococcal conjugates vaccines^48^. Herein, the significant correlation between the magnitude of the anti-SF2a LPS IgG response following SF2a-TT15 administration and SBA titers at 3 months post-vaccination demonstrates the functional capabilities of the anti-SF2a LPS IgG antibodies induced by this conjugate^39^. In line with the above, SBA was also successfully used to document the functionality of serum antibodies elicited by other *Shigella* candidate vaccines in recent phase I, IIa and IIb clinical trials^26,49–52^. However, to the best of our knowledge, our study is the first to examine the long-term durability of SBA induced by a *Shigella* vaccine candidate.

Interestingly, there was a good to an excellent correlation between the levels of humoral parameters measured at a relatively short time (3 months) after vaccination with a 3-injection regimen with those measured 2 and 3 years later. This suggests that the magnitude and functionality of the short-term immune responses induced by SF2a-TT15 could predict their longevity. This finding could be extended and further confirmed in the case of other vaccine candidates targeting an anti-bacterial LPS immune response.

To the best of our knowledge, our study is also the first one that evaluated the long-term durability of memory B-cell response up to 2 and 3 years after immunization with a *Shigella* vaccine candidate. In contrast to the relatively long persistence of the humoral immune response induced by SF2a-TT15, the persistence of circulating memory IgG and IgA B-cell specific to SF2a LPS as measured by the ELISPOT assay was shorter, declining toward pre-immunization levels 2 and 3 years later. The persistence of memory B-cells in peripheral blood is agent and antigen-dependent. Available data suggest that it is usually longer following viral as compared to bacterial infections or protein versus polysaccharide antigen stimulation^53,54^. While not detectable in circulation, the antigen-specific memory B-cells may reside in secondary lymphoid organs or in the bone marrow, as it has been suggested^55,56^, ready to quickly proliferate and differentiate into antibody-producing plasma cells upon antigen re-exposure. In a recent study performed on an oral multivalent ETEC vaccine, it was shown that a mucosal memory response was sustained for 13–23 months post-vaccination. This was indicated by an anamnestic response to a single booster dose given 13–23 months later, while circulating vaccine-specific IgA memory B-cells were undetectable and circulating specific-IgG memory B-cells were found only to one (out of five) of vaccine components at this point of time^57^. These data and those of our study corroborate suggesting that measuring circulating memory B-cells in peripheral blood at a long period of time after exposure to a bacterial antigen might not be an alternative to assessing an anamnestic response after boosting in order to document immunological memory.

An important finding of our study is the association between the percent of specific-IgG antibody-secreting cells detected by ELISPOT 3 months after vaccination and the persistence of the humoral immune response components 2 years later. Vaccinees who exhibited specific-IgG memory B-cell levels above the median at 3 months post-last vaccination had a better chance to maintain high levels of the humoral parameters tested at least 2 years post-vaccination. This suggests that memory B-cell counts measured within a few months after vaccination with the SF2a-TT15 vaccine candidate could also be a predictor of the durability of the specific humoral antibody response although themselves return to pre-vaccination levels 2–3 years later.

One may raise the possibility that the pre-existing levels of O-SP-binding and functional antibodies among volunteers suggest pre-exposure to SF2a and that the glycoconjugate has boosted immunity obtained through natural exposure. We consider unlikely that volunteers were exposed to SF2a prior to their enrollment in the phase I study. We rather assume that the pre-existing levels of antibodies are induced and maintained following encounters with cross-reacting antigens of other Enterobacteriaceae during life span as has been shown in various populations^20,24^. We minimized by different ways the chance that volunteers were pre-exposed to SF2a or could have been exposed during the phase I trial and follow-up. On the one hand, history of culture-proven *S. flexneri* shigellosis or traveling in areas endemic for *S. flexneri* within 3 months before enrollment were exclusion criteria for participation in the study. On the other hand, the study volunteers were recruited from the center of Israel and the Tel Aviv area where the risk of exposure to *S. flexneri* is extremely low. Not the least, volunteers were pre-screened for low anti-SF2a-LPS serum IgG levels^39^. Of note, along the 3 years of follow up, there were no significant changes in the immunological parameters among placebo recipients, suggesting the absence of natural exposure of the volunteers to SF2a O-SP or any cross-reacting antigens during this period. Moreover, none of the volunteers exhibited higher levels of serum IgG or IgA or antibody functionality 2 and 3 years after vaccination than those observed 3 months after vaccination.

In conclusion, the SF2-TT15 synthetic carbohydrate-based vaccine candidate induced a long-lasting immune response in adults in a high-income country. Similar follow-up studies are advised in children/infants involved in the ongoing/planned *Shigella* vaccine phase IIa trials in LMICs assessing also the contribution of concomitant natural exposure. Otherwise, added to the original data of phase I clinical trial, the outcome of the present long-term follow-up study strongly supports the further evaluation of SF2a-TT15 for safety and immunogenicity in infants in LMICs and for preliminary efficacy in a CHIM study in North American adult volunteers. Toward this end, both the 2 µg and 10 µg OS doses of SF2a-TT15, adjuvanted or non-adjuvanted, are currently evaluated in young children and 9-month-old infants in Kenya (ClinicalTrials.gov Identifier: NCT04602975), while the 10 µg OS alum-adjuvanted vaccine dose is used in the ongoing CHIM study in North American volunteers (ClinicalTrials.gov Identifier: NCT04078022)^58^.

Moreover, as for the other most advanced LPS-based *Shigella* vaccine candidates, a broad serotype coverage *Shigella* vaccine candidate featuring a suitable combination of rationally designed fine-tuned synthetic OS representative of segments from the O-SPs of selected *S. flexneri* serotypes and *S. sonnei* is currently in pre-clinical development^58^.

Further development will be guided by data from the current phase IIa clinical trial of SF2a-TT15 in infants in Kenya. In particular, it is possible that in the infant target population in LMICs the immune response could be of a lower magnitude than that achieved in the phase I study. If so, its enhancement by appropriate adjuvant manipulation would have to be considered. Otherwise, providing confidence in this promising strategy is the natural oral priming at the age of 9 months targeted for the first dose of a *Shigella* vaccine in LMICs that was recently documented by the observed early rise in anti-SF2a and *S. sonnei* LPS antibodies in a meaningful proportion of Zambian children aged 14–52 weeks^59^. This important finding may play a supportive role in the immune response and subsequent protection following the parenteral delivery of the highly immunogenic SF2a-TT15 vaccine candidate.

Similar considerations could be true for the other most advanced LPS-based *Shigella* vaccine candidates. However, to evaluate the protective efficacy against shigellosis in infants in LMICs, one has to wait for a phase III trial that will also constitute the most suitable setup to confirm the correlate of protection status for O-SP IgG in these settings. This could also further facilitate the licensure of subsequent vaccines on the basis of safety, immunogenicity and non-inferior immunogenicity^19,60^.

## Methods

### Follow-up

**Fig. 5:**
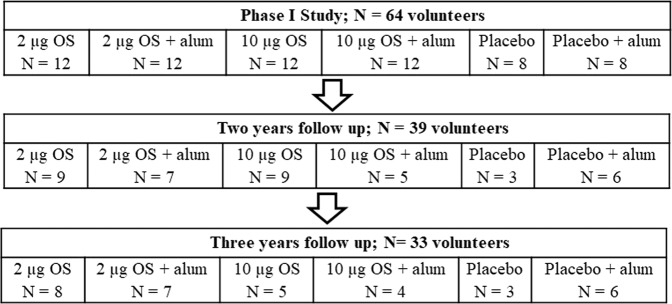
Upper panel (phase I study), middle panel (at 2-year follow-up) and lower panel (at 3-year follow-up). Distribution of volunteers in the long-term follow up according to the randomized allocation in the phase I study.

The follow up included two visits of volunteers at the Clinical Research Center, Tel Aviv Sourasky Medical Center at two time points corresponding approximately to 2 and 3 years after the last vaccine injection. All 64 volunteers of the phase I study were approached by phone by the study team. From the volunteers participating in the phase I study, 39 subjects were enrolled successfully in the first follow-up visit between May and June 2019, and 33 of them in the second visit, between July and August 2020. The subject distribution in the long-term follow-up according to the randomized allocation in the phase I study is given in Fig. 5. Reasons of the rest of the participants in the phase I study for not enrolling in the follow-up included: not being available by phone despite multiple attempts, unwilling to participate for various reasons, in active military service or living abroad. Answering a short questionnaire, none of the subjects had any recollection or knowledge of exposure to *Shigella* in the time that elapsed between vaccination and follow-up visits. Written informed consent was obtained from each participant before enrollment. The study protocol was reviewed and approved by the Tel Aviv Sourasky Medical Center Institutional Ethics Committee and by the Tel Aviv University Ethical Committee.

### Sample collection

Whole blood and serum samples were obtained from volunteers at each visit to the Clinical Research Center of Tel Aviv Sourasky Medical Center and transferred within 2 hours of collection to the School of Public Health Research Lab, at the Sackler Faculty of Medicine, Tel Aviv University for further processing and storage. Peripheral blood mononuclear cells (PBMC) were isolated from whole blood followed by activation or cryopreservation. Serum samples were centrifuged within 4 hours after collection and stored at -80 °C until further analyzed. The assay procedures and analyses of all parameters described below were identical in the phase I and follow-up studies. Samples of volunteers collected in the phase I study were retested in the same runs with the new samples obtained during the follow up. The results obtained at retesting were in all cases below 2-fold change in titer compared to the original data.

### Measurement of serum IgG, IgA to SF2a LPS by ELISA

Serum IgG and IgA antibodies were measured by an ELISA in-house protocol. Briefly, 96 well microtiter plates (Corning) were coated with 5 µg/mL of SF2a LPS (extracted from SF2a 454) in carbonate buffer for 1 h at 37 °C. Unbound sites were blocked with 150 µl of blocking buffer containing 0.5% bovine serum albumin (BSA) (Merck Millipore Corp.) and 0.5% Casein for 1 h at 37 °C. Wells were washed twice (PBS Tween) and duplicates of tested samples were serially 2-fold diluted (initial dilution 1:200) in blocking buffer, added to the wells and incubated overnight (ON) at room temperature (RT). Plates were washed four times and 100 µl/well of alkaline phosphatase (AP) conjugated anti-human IgG or IgA (KPL, Inc. USA) was added and incubated ON at RT. Wells were washed four times and 100 µl/well of phosphatase substrate, *para*-nitrophenyl phosphate (pNPP) one component (SouthernBiotech) was added and plates were incubated in the dark for 15 min at RT. The reaction was stopped by the addition of 50 µl/well of Stop solution (3 M NaOH). Absorbance was measured at 405 nm using an ELISA plate reader (Multiskan FC, Thermo Scientific). Results were expressed in endpoint titers (the last serum dilution yielding an optical density (O.D.) of 0.2 or higher).

### Serum bactericidal activity

SBA was measured by a quantitative in-house titration method^52,61^. Heat-inactivated serum samples were serially 2-fold diluted (initial dilution 1:50) in phosphate-buffered saline (PBS) in 96 U-shape microtiter plates (Greiner-bio-one International) to a final volume of 40 µL/well. A bacterial suspension of SF2a 2457 T was grown in Tryptic Soy Broth (Beckton, Dickinson) supplemented with 0.4% yeast extract at 37 °C in a shaker incubator to O.D 0.25-0.3 at 600 nm (~7 × 10^7^ cfu/mL). The suspension was diluted 1:10,000 and 40 µL/well were added to the microtiter plates. Plates were placed in a shaker incubator at 37 °C for 15 min followed by the addition of 20 µL/well of Baby Rabbit Complement (BRC) (CEDERLANE Corp.) and 1 h incubation at 37 °C without shaking. 20 µl of the samples from each well were diluted in 380 µl of PBS followed by plating six times 20 µL drops on Tryptic Soy Agar-Congo Red plates (Hylabs). The number of colonies was counted and the percent of bacterial survival compared to control wells (containing bacteria and BRC without serum) was calculated. A serum yielding at least 50% reduction in the number of colonies compared to control wells was defined as having bactericidal activity. The last serum dilution yielding bactericidal activity was defined as end point titer.

### Antibody avidity

Antibody avidity (functional affinity), defined as the overall binding strength of an antibody to an antigen was measured by competitive inhibition^45^. Briefly, 96 well microtiter plates were coated with 5 µg/mL of SF2a LPS incubated for 1 h at 37 °C and blocked with 150 µl of blocking buffer (containing 0.5% BSA and 0.5% Casein) for another 1 h. Double dilutions of SF2a LPS, here as free antigen, in blocking buffer were added to the microtiter plate (initial concentration 5 µg/mL). The last well was covered only with blocking buffer (control well). Sera at O.D. of approx. 1.0 at A_405_ were added to the wells containing free antigen or blocking buffer and plates were incubated ON. After washing plates six times, 100 µl of AP-conjugated anti-human IgG (KPL Inc. USA) was added followed by ON incubation. Wells were washed four times, 100 µl of pNPP one component substrate was added and the plates were incubated in the dark for 15 min at RT. Plates were read at 405 nm until the O.D. of control wells reaches 0.8 to 1.2. The results of relative avidity, avidity index (AI), represent the free antigen concentration that was required to inhibit antibody binding to the coated SF2a LPS wells by 50%. This value could also be expressed as the log of the free antigen concentration that is necessary to cause 50% inhibition (*I*_50_).

### Activation and measurement of IgG and IgA memory B-cells

Activation of PBMC and ELISPOT assay was performed for the measurement of IgG and IgA memory B-cell response to SF2a LPS^62,63^. PBMCs were isolated from whole blood using Ficoll-Paque PLUS (GE Healthcare) and incubated for 5 days with the activating reagents 10 ng/mL IL-2 (R&D Systems) and 1 µg/mL R848 (Invivogen) in RPMI-1640 supplemented with 10% Fetal Bovine Serum (BI inc, Rhenium). ELISPOT PVDF microtiter plates (Merck Millipore Corp.) were coated with 5 µg/mL of SF2a LPS or with Goat anti-human IgG-Fc antibody (EMD Millipore Corp.) or Goat anti-human IgA antibody (Bethyl Laborathories Inc.). Activated PBMCs were washed and added in duplicates in two concentrations (3 × 10^5^/100 µL/well and 1.5 × 10^5^/100 µL/well) to LPS coated wells. Activated PBMC at 3 × 10^3^/100 µL/well were serially 2-fold diluted and added to anti-IgG/IgA coated wells. Plates were incubated in a 37 °C CO_2_ humidified incubator for 6 h and washed twice with PBS-Tween and twice with PBS. AP labeled anti-human IgG or anti-human IgA goat antibodies diluted 1:4000 (Bethyl Laborathories Inc.) were added to the wells and incubated ON at 4 °C. Plates were washed twice with PBS-Tween, twice with PBS, once with double distilled water (ddw) and the BCIP/NBT (Merck Millipore Corp.) substrate solution was added and incubated for 20 min. Reaction was stopped by washing twice with ddw and incubating with 200 µL/well ddw for 30 min in RT. The number of spots was counted using a stereoscope attached to a camera (SMZ800N, Nikon). Results are expressed as % LPS-specific antibody-secreting cells/total IgG or IgA secreting cells. An individual positive response after vaccination was defined as an increase in the percentage of SF2a LPS-specific-IgG and IgA secreting cells per total IgG or IgA secreting cells above a cut-off value represented by the mean of the same parameter and two Standard Deviations (SDs) calculated among all placebo recipients at each time point.

### Statistical analysis

Geometric mean (GM), geometric mean titer (GMT) and 95% Confidence Intervals (CIs) were calculated for immunological parameters and summarized by time after vaccination. The percentages of responders were determined by defining a priori cut-off values for each immunological parameter: for serum IgG, IgA and SBA the cut-off value was ≥4-fold rise in titer compared to baseline, for memory B cells the cut-off value was mean + 2 SD of all placebo recipients at each time point.

Differences between GMTs or GMs at follow up versus pre-vaccination and 3 months post-last injection time points were examined by the Wilcoxon signed rank test for repeated measurements. Correlations between immunological parameters were assessed using Spearman’s rank correlation coefficient. Fisher’s exact test was used to assess unadjusted associations of the frequency of specific-IgG or IgA memory B-cells, as categorical variables, with the percentage of responders for serum IgG, IgA and SBA, as well as Student’s *t* test for the association with mean avidity. All statistical tests were interpreted in a two-tailed fashion using a significance level (α) = 0.05. Data were analyzed using the SPSS version 24 (Armok, N.Y., USA).

### Reporting summary

Further information on research design is available in the Nature Research Reporting Summary linked to this article.

## Supplementary information

Supplemental material

REPORTING SUMMARY

## Data Availability

The authors declare that the main data supporting the findings of this study are available within the article and its Supplementary Material file. Extra aggregative data can be made available upon a request from the corresponding author.
