# Supplementary material for: A Shigella flexneri 2a synthetic glycan-based vaccine induces a long-lasting immune response in adults
Source: NPJ Vaccines. 2023 Mar 10;8:35. doi: 10.1038/s41541-023-00624-y (PMC9998260; doi:10.1038/s41541-023-00624-y)
Supplement: Supplementary file 1 — Supplemental material [file 41541_2023_624_MOESM1_ESM.pdf]

Supplementary Material

Supplementary Figure 1. Longevity of serum IgA GMT (95% CI) to SF2a LPS in vaccinees receiving 2 or 10 µg OS doses of non-adjuvanted and adjuvanted SF2a-TT15 and in placebo recipients. Circles represent individual endpoint titres, green circles represent volunteers receiving adjuvanted vaccine or placebo. Bars represent the Geometric Mean Titer (GMT) and the 95% Confidence Intervals (CIs) on day 0 (baseline), three months, two and three years post last vaccination.

*****p-value <0.05; ******p-value <0.01 (vs. day 0) Wilcoxon signed rank test.

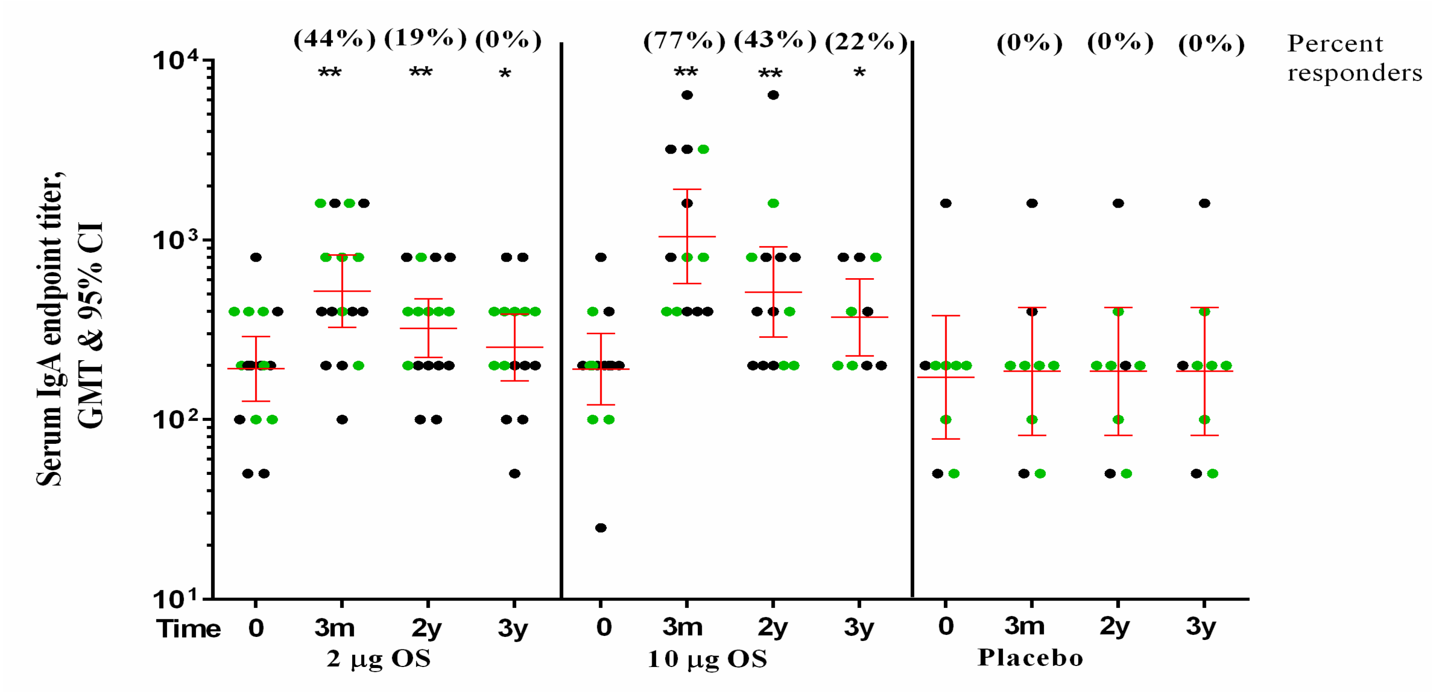

Supplementary Figure 2: Longevity of IgA memory B-cells to SF2a LPS in vaccinees receiving non-adjuvanted and adjuvanted 2 or 10 µg OS doses of SF2a-TT15 and in placebo recipients. Circles represent individual percent of IgA antibody secreting cells (ASCs) to SF2a LPS /Total IgA ASCs, green circles represent volunteers receiving adjuvanted vaccine or placebo. Bars represent the Geometric Mean (GM) and 95% Confidence intervals (CIs) of these values on day 0 (baseline), three months, two and three years post-vaccination.

***** p-value <0.05; ****** p-value <0.01 (vs. day 0) Wilcoxon signed rank test.

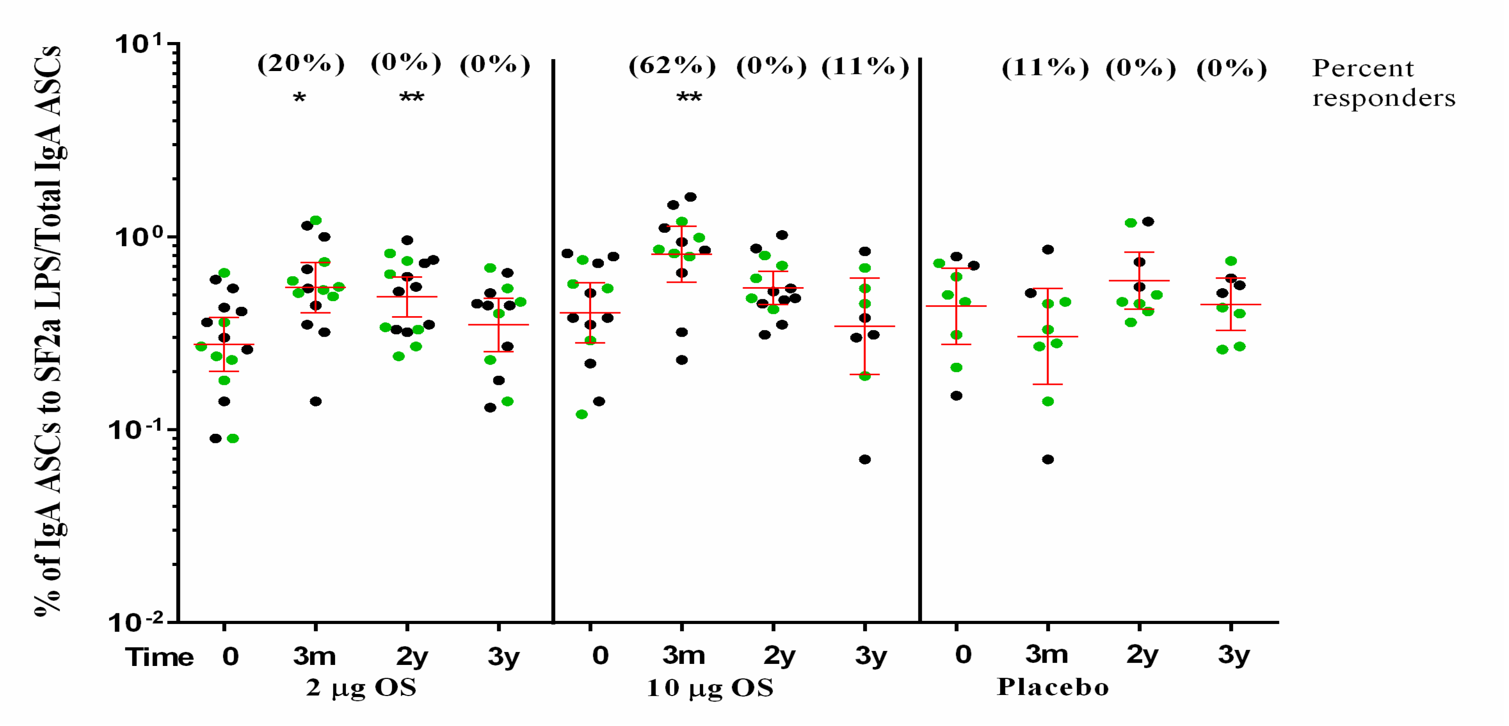

Supplementary Table 1: Serum IgG, SBA, avidity and serum IgA responses to SF2a LPS in vaccinees receiving non-adjuvanted and adjuvanted 2 or 10 µg OS doses of SF2a-TT15 and in placebo recipients.

Parameter	Treatment	N	day 0 (baseline) GMT (95% CI)	3 months after vaccination GMT (95% CI), % responders	2 years after vaccination GMT (95% CI), % responders	N	3 years after vaccination GMT (95% CI), % responders
Serum IgG (responder: ≥ 4-fold rise in titer)	2µg	9	370 (199-690)	1372 (766-2455), 55.6	800 (470-1363), 33.3	8	734 (506-1064), 25
	2µg+alum	7	594 (263-1344)	3533 (1288-9688), 71.4	1188 (526-2688), 42.9	7	975 (530-1794), 14.3
	2µg total	16	456 (290-716)	2075# (1212-3551), 10/16=62.5	951* (628-1442), 6/16=37.5	15	838* (617-1138), 3/15=20
	10µg	9	467 (299-727)	6979 (2448-19895), 87.5	2177 (1019-4650), 77.8	5	2111 (791-5632), 80
	10µg+alum	5	460 (179-1180)	12800 (4461-36728), 100	3200 (821-12478), 100	4	2263 (545-9398), 100
	10µg total	14	464 (325-663)	8813# (4484-17321), 12/13=92.3	2498# (1400-4458), 12/14=85.7	9	2177# (1192-3976), 8/9=88.9
	placebo	9	370 (244-562)	343 (240-489), 0	370 (211-649), 0	9	343 (181-651), 0
SBA (responder: ≥ 4-fold rise in titer)	2µg	9	233 (60-902)	2540 (379-17023), 77.8	1372 (279-6749), 66.7	8	872 (183-4161), 50
	2µg+alum	7	135 (32-561)	1950 (201-18934), 57.1	1189 (163-8681), 57.1	7	800 (126-5091), 42.9
	2µg total	16	183 (77-439)	2263# (634-8076), 11/16=68.8	1288# (435-3813), 10/16=62.5	15	838# (299-2348), 7/15=46.7
	10µg	9	185 (46-786)	7611 (2757-21013), 100	3200 (1506-6798), 66.7	5	2425 (453-12983), 40
	10µg+alum	5	348 (30-4094)	2111 (65-68170), 60	1600 (76-33545), 60	3	400 (1-198743), 33.3
	10µg total	14	232 (79-682)	4648# (1411-15313), 11/13=84.6	2498# (992-6291), 9/14=64.3	8	1234* (247-6158), 3/8=37.5
	placebo	9	544 (120-2463)	504 (123-2064), 0	544 (129-2291), 0	8	238 (43-1325), 0
Avidity (Mean I_{50})	2µg	9	2.43 (1.87-3)	1.89 (1.69-2.08)	1.84 (1.45-2.24)	8	2.03 (1.56-2.5)
	2µg+alum	7	2.45 (1.81-3.09)	1.65 (1.36-1.95)	2.05 (1.45-2.65)	7	2.05 (1.44-2.65)
	2µg total	16	2.44 (2.07-2.81)	1.78#(1.63-1.94)	1.93# (1.63-2.23)	15	2.04# (1.72-2.36)
	10µg	9	2.31 (1.82-2.79)	1.69 (1.33-2.05)	1.78 (1.47-2.09)	5	2.03 (1.86-2.2)
	10µg+alum	5	2.83 (2.34-3.31)	1.7 (1.48-1.91)	1.9 (1.64-2.16)	4	1.89 (1.79-1.98)
	10µg total	14	2.49 (2.15-2.84)	1.69# (1.47-1.91)	1.82# (1.62-2.02)	9	1.97# (1.87-2.07)
	placebo	9	2.58 (2.31-2.85)	2.57 (2.17-2.97)	2.41 (2.15-2.68)	9	2.36 (2.07-2.65)
Serum IgA (GMT) (responder: ≥ 4-fold rise in titer)	2µg	9	171 (86-343)	400 (198-809), 33.3	272 (141-526), 11.1	8	200 (88-454), 0
	2µg+alum	7	221 (124-393)	725 (365-1438), 57.1	400 (276-579), 28.6	7	328 (240-449), 0
	2µg total	16	192 (126-290)	519# (326-826), 7/16=43.8	322# (221-469), 3/16=18.8	15	252* (164-386), 0/15=0
	10µg	9	200 (99-405)	1234 (489-3115), 75	544 (233-1270), 55.6	5	400 (169-946), 40
	10µg+alum	5	174 (85-358)	800 (279-2296), 80	460 (150-1411), 20	4	336 (117-967), 0
	10µg total	14	190 (121-301)	1044# (570-1914), 10/13=76.9	512# (287-914), 6/14=42.9	9	370* (226-607), 2/9=22.2
	placebo	9	171 (78-378)	185 (82-420), 0	185 (82-420), 0	9	185 (82-420), 0

*p<0.05 versus day 0 (baseline); #p<0.01 versus day 0 (baseline) by Wilcoxon signed rank test

Abbreviations: serum bactericidal antibodies (SBA), Geometric Mean Titer (GMT), Confidence interval (CI).

Supplementary Table 2: IgG and IgA memory B-cell responses to SF2a LPS in vaccinees receiving non-adjuvanted and adjuvanted 2 or 10 µg OS doses of SF2a-TT15 and in placebo recipients.

Parameter	Treatment	N	day 0 (baseline) GM (95% CI)	3 months after vaccination GM (95% CI), % responders	2 years after vaccination GM (95% CI), % responders	N	3 years after vaccination GM (95% CI), % responders
IgG Memory B-cell response (GM of % ASCs) (responder: ≥ mean+2SD)	2µg	9	0.01 (0-0.04)	0.04 (0.01-0.09), 37.5	0.01 (0-0.03), 0	8	0.01 (0-0.03), 25
	2µg+alum	7	0.01 (0-0.04)	0.03 (0.01-0.12), 14.3	0.01 (0-0.05), 0	7	0.01 (0-0.04), 14.3
	2µg total	16	0.01 (0-0.02)	0.03* (0.02-0.07), 4/15=26.7	0.01 (0-0.02), 0/16=0	15	0.01 (0-0.02), 1/15=6.7
	10µg	9	0.02 (0.01-0.06)	0.1 (0.07-0.14), 75	0.03 (0.02-0.05), 11.1	5	0.01 (0-0.05), 20
	10µg+alum	5	0.01 (0-0.11)	0.1 (0.03-0.29), 60	0.03 (0.01-0.07), 20	4	0.04 (0.02-0.11), 75
	10µg total	14	0.02 (0.01-0.04)	0.1[#] (0.07-0.14), 9/13=69.2	0.03 (0.02-0.04), 2/14=14.3	9	0.01 (0-0.05), 1/9=11.1
	placebo	9	0.01 (0-0.02)	0.02 (0.01-0.04), 0	0.01 (0-0.03), 0	7	0 (0-0.01), 0
IgA Memory B-cell response (GM of % ASCs) (responder: ≥ mean+2SD)	2µg	9	0.3 (0.19-0.49)	0.48 (0.27-0.85), 25	0.53 (0.39-0.72), 0	8	0.34 (0.21-0.54), 0
	2µg+alum	7	0.25 (0.14-0.43)	0.63 (0.47-0.85), 14.3	0.43 (0.27-0.69), 0	6	0.36 (0.19-0.67), 0
	2µg total	16	0.28 (0.2-0.38)	0.55* (0.4-0.74), 3/15=20	0.49[#] (0.38-0.62), 0/16=0	14	0.35 (0.25-0.48), 0/14=0
	10µg	9	0.42 (0.26-0.66)	0.75 (0.42-1.34), 62.5	0.52 (0.39-0.7), 0	5	0.29 (0.1-0.89), 20
	10µg+alum	5	0.38 (0.15-0.95)	0.92 (0.74-1.14), 60	0.59 (0.42-0.82), 0	4	0.42 (0.17-1.03), 0
	10µg total	14	0.4 (0.28-0.58)	0.81[#] (0.58-1.13), 8/13=61.5	0.54 (0.45-0.66), 0/14=0	9	0.34 (0.19-0.61), 1/9=11.1
	placebo	9	0.44 (0.28-0.69)	0.3 (0.17-0.54), 11.1	0.59 (0.42-0.83), 0	8	0.45 (0.33-0.61), 0

*p<0.05 versus day 0 (baseline); #p<0.01 versus day 0 (baseline) by Wilcoxon signed rank test; Abbreviations: Geometric Mean (GM), Confidence interval (CI).

Supplementary Table 3: Frequency of specific IgA memory B-cells at three months post last vaccination (with 2 or 10 µg OS doses of SF2a-TT15 non-adjuvanted and adjuvanted) defined as "low" and "high" values around the geometric mean, and percent of responders (≥4-fold) for serum IgG, serum IgA, SBA and mean value for avidity against SF2a LPS, two years after vaccination.

Parameters 2 years after vaccination		Low frequency (≤0.55) N=11	High frequency (>0.55) N=17	p value
Serum IgG	≥ 4-fold	7 (63.6%)	10 (58.8%)	1.000*
Serum IgA	≥ 4-fold	4 (36.4%)	5 (29.4%)	1.000*
SBA	≥ 4-fold	7 (63.6%)	11 (64.7%)	1.000*
Avidity (I_{50})	n (Mean)	11 (1.77)	17 (1.96)	0.307#

Geometric mean of IgA memory B-cells ASCs three months after vaccination = 0.55.

*Fisher's exact test; #Student t test.

Abbreviations: serum bactericidal antibodies (SBA), Antibody secreting cells (ASCs), Avidity index: I_{50}.
