# Supplementary material for: A Shigella flexneri 2a synthetic glycan-based vaccine induces a long-lasting immune response in adults
Source: NPJ Vaccines. 2023 Mar 10;8:35. doi: 10.1038/s41541-023-00624-y (PMC9998260; doi:10.1038/s41541-023-00624-y)
Supplement: Supplementary file 2 — REPORTING SUMMARY [file 41541_2023_624_MOESM2_ESM.pdf]

nature portfolio

Corresponding author(s): Dani Cohen
Last updated by author(s): Jan 7, 2023

Reporting Summary

Nature Portfolio wishes to improve the reproducibility of the work that we publish. This form provides structure for consistency and transparency in reporting. For further information on Nature Portfolio policies, see our Editorial Policies and the Editorial Policy Checklist.

Statistics

For all statistical analyses, confirm that the following items are present in the figure legend, table legend, main text, or Methods section.

n/a	Confirmed	
☐	☒	The exact sample size (*n*) for each experimental group/condition, given as a discrete number and unit of measurement
☐	☒	A statement on whether measurements were taken from distinct samples or whether the same sample was measured repeatedly
☐	☒	The statistical test(s) used AND whether they are one- or two-sided<br>*Only common tests should be described solely by name; describe more complex techniques in the Methods section.*
☐	☒	A description of all covariates tested
☐	☒	A description of any assumptions or corrections, such as tests of normality and adjustment for multiple comparisons
☐	☒	A full description of the statistical parameters including central tendency (e.g. means) or other basic estimates (e.g. regression coefficient) AND variation (e.g. standard deviation) or associated estimates of uncertainty (e.g. confidence intervals)
☐	☒	For null hypothesis testing, the test statistic (e.g. *F*, *t*, *r*) with confidence intervals, effect sizes, degrees of freedom and *P* value noted<br>*Give P values as exact values whenever suitable.*
☒	☐	For Bayesian analysis, information on the choice of priors and Markov chain Monte Carlo settings
☒	☐	For hierarchical and complex designs, identification of the appropriate level for tests and full reporting of outcomes
☐	☒	Estimates of effect sizes (e.g. Cohen's *d*, Pearson's *r*), indicating how they were calculated

Our web collection on statistics for biologists contains articles on many of the points above.

Software and code

Policy information about availability of computer code

Data collection	no software was used in data collection
Data analysis	Data were analysed using the SPSS version 24 (Armok, N.Y., USA). © 2021 GraphPad Prism Software, Inc was emplyed for graphical display of results

For manuscripts utilizing custom algorithms or software that are central to the research but not yet described in published literature, software must be made available to editors and reviewers. We strongly encourage code deposition in a community repository (e.g. GitHub). See the Nature Portfolio guidelines for submitting code & software for further information.

Data

Policy information about availability of data

All manuscripts must include a data availability statement. This statement should provide the following information, where applicable:

- Accession codes, unique identifiers, or web links for publicly available datasets
- A description of any restrictions on data availability
- For clinical datasets or third party data, please ensure that the statement adheres to our policy

The main data supporting the findings of this study are available within the article and its Supplementary Material file. Extra aggregative data can be made available upon a request from the corresponding author.

Human research participants

Policy information about studies involving human research participants and Sex and Gender in Research.

Reporting on sex and gender	See below
Population characteristics	See below
Recruitment	See below
Ethics oversight	Written informed consent was obtained from each participant before enrolment. The study protocol was reviewed and approved by the Tel Aviv Sourasky Medical Center Institutional Ethics Committee and by the Tel Aviv University Ethical Committee.

Note that full information on the approval of the study protocol must also be provided in the manuscript.

Field-specific reporting

Please select the one below that is the best fit for your research. If you are not sure, read the appropriate sections before making your selection.

☐ Life sciences ☒ Behavioural & social sciences ☐ Ecological, evolutionary & environmental sciences

For a reference copy of the document with all sections, see nature.com/documents/nr-reporting-summary-flat.pdf

Behavioural & social sciences study design

All studies must disclose on these points even when the disclosure is negative.

Study description	Follow-up study to examine the longevity of the immune response among the volunteers of a phase I clinical trial (completed in December 2017) of an investigational conjugate vaccine (SF2a-TT15) against shigellosis, two and three years after vaccination. Quantitative data were collected on paramenters related to the longevity of the immune response to the investigational vaccine.
Research sample	39 volunteers (29 males and 10 females, aged 18-45) were enroled successfully in the first follow-up visit (2 years after vaccination) and 33 of them in a second visit (3 years after vaccination).
Sampling strategy	All 64 participants of the phase I study were approached by phone by the study team to volunteer to a follow-up study to examine the longevity of the immune response to the investigational vaccine. 39 subjects were enrolled successfully in the first follow up visit (2 years after vaccination) and 33 of them in the second visit (3 years after vaccination).
Data collection	A short questionnaire, including questions on potential exposure to Shigella in the time that elapsed between vaccination and follow-up visits.
Timing	First follow-up visit, between May and June 2019, and 3 second follow-up visit, between July and August 2020.
Data exclusions	No exclusions of follow-up data
Non-participation	Reasons of the rest of the participants in the phase I study for not enrolling in the follow up included: not being available by phone despite multiple attempts, unwilling to participate for various reasons, in active military service or living abroad. Of note, both follow-up visits were conducted between the waves/lockdowns of the COVID-19 epidemic in Israel.
Randomization	Participants belonged to the experimental groups of the phase I study to which they were randomized in 2016-2017 (2 and 3 years before the follow-up study).

Reporting for specific materials, systems and methods

We require information from authors about some types of materials, experimental systems and methods used in many studies. Here, indicate whether each material, system or method listed is relevant to your study. If you are not sure if a list item applies to your research, read the appropriate section before selecting a response.

Materials & experimental systems

n/a	Involved in the study	
☒	☐	Antibodies
☒	☐	Eukaryotic cell lines
☒	☐	Palaeontology and archaeology
☒	☐	Animals and other organisms
☐	☒	Clinical data
☒	☐	Dual use research of concern

Methods

n/a	Involved in the study	
☒	☐	ChIP-seq
☒	☐	Flow cytometry
☒	☐	MRI-based neuroimaging

Clinical data

Policy information about clinical studies

All manuscripts should comply with the ICMJE guidelines for publication of clinical research and a completed CONSORT checklist must be included with all submissions.

Clinical trial registration

Not applicable. The current study was not a clinical/intervention trial but a longterm immunological follow-up study on persistence of immunological parameters following immunization with a Shigella investigational vaccine in frame of a completed phase I study (ClinicalTrials.gov Identifier: NCT02797236).

Study protocol

The follow-up study protocol approved by the Tel Aviv Sourasky Medical Center Institutional Ethics Committee and by the Tel Aviv University Ethical Committee can be accessed through the Principal Investigator and Scientific Leader of the study.

Data collection

A short questionnaire, including questions on potential exposure to Shigella in the time that elapsed between vaccination and follow-up visits. Blood samples and urine samples were collected from volunteers at the follow-up visits 2 and 3 years after vaccination in frame of the completed phase I study.

Outcomes

The outcomes of the study included only immunological parameters charecterizing the durability of the specific immune response to S. flexneri 2a LPS 2 and 3 years after vaccination.
